# The elephant in the room: Exploring the influence and participation of patients in infection‐related care across surgical pathways in South Africa and India

**DOI:** 10.1111/hex.13715

**Published:** 2023-01-31

**Authors:** Vrinda Nampoothiri, Oluchi Mbamalu, Surya Surendran, Candice Bonaconsa, Timothy Pennel, Adam Boutall, Kirun Gopal, Enrique Castro Sanchez, Puneet Dhar, Alison Holmes, Sanjeev Singh, Marc Mendelson, Carolyn Tarrant, Esmita Charani, ASPIRES Co‐Investigators

**Affiliations:** ^1^ Department of Infection Control and Epidemiology Amrita Institute of Medical Science, Amrita Vishwa Vidyapeetham Kochi Kerala India; ^2^ Department of Medicine, Division of Infectious Diseases & HIV Medicine, Groote Schuur Hospital University of Cape Town Cape Town South Africa; ^3^ Division of Health System and Equity The George Institute for Global Health New Delhi India; ^4^ Division of Cardiothoracic Surgery University of Cape Town Cape Town South Africa; ^5^ Colorectal Unit, Groote Schuur Hospital University of Cape Town Cape Town South Africa; ^6^ Department of Cardiovascular and Thoracic Surgery Amrita Institute of Medical Sciences, Amrita Vishwa Vidyapeetham Kochi Kerala India; ^7^ School of Health Sciences, Division of Nursing University of London London UK; ^8^ National Institute for Health and Care Research, Health Protection Research Unit in Healthcare Associated Infection and Antimicrobial Resistance Imperial College London London UK; ^9^ All India Institute of Medical Sciences Rishikesh India; ^10^ Department of Health Sciences University of Leicester Leicester UK; ^11^ Faculty of Health and Life Sciences University of Liverpool Liverpool UK

**Keywords:** antibiotic use, antimicrobial stewardship, ethnography, patient carer, patient involvement, patient roles

## Abstract

**Objective:**

The irrational use of antibiotics is a leading contributor to antibiotic resistance. Antibiotic stewardship (AS) interventions predominantly focus on prescribers. This study investigated the influence and participation of inpatients in infection‐related care, including antibiotic decision‐making, within and across two tertiary hospitals in South Africa (Cape Town) and India (Kerala).

**Methods:**

Through ethnographic enquiry of clinical practice in surgical pathways, including direct nonparticipant observation of clinical practices, healthcare worker (HCW), patient and carer interactions in surgical ward rounds and face‐to‐face interviews with participants (HCWs and patients), we sought to capture the implicit and explicit influence that patients and carers have in infection‐related care. Field notes and interview transcripts were thematically coded, aided by NVivo 12® Pro software.

**Results:**

Whilst observational data revealed the nuanced roles that patients/carers play in antibiotic decision‐making, HCWs did not recognize these roles. Patients and carers, though invested in patient care, are not routinely involved, nor are they aware of the opportunities for engagement in infection‐related decision‐making. Patients associated clinical improvement with antibiotic use and did not consider hospitalization to be associated with infection acquisition or transmission, highlighting a lack of understanding of the threat of infection and antibiotic resistance. Patients' economic and cultural positionalities may influence their infection‐related behaviours. In the study site in India, cultural norms mean that carers play widespread but unrecognized roles in inpatient care, participating in infection prevention activities.

**Conclusion:**

For patients to have a valuable role in AS and make informed decisions regarding their infection‐related care, a mutual understanding of their role in this process among HCWs and patients is crucial. The observed differences between the two study sites indicate the critical need for understanding and addressing the contextual drivers that impact effective patient‐centred healthcare delivery.

**Patient or Public Contribution:**

Ethnographic observations and interviews conducted in this study involved patients as participants. Patients were recruited for interviews after obtaining signed informed consent forms. Patients' identities were completely anonymized when presenting the study findings.

## 1 INTRODUCTION

Antibiotic use in human populations remains a key driver of antibiotic resistance (ABR) worldwide.^1^, ^2^ Unregulated access to antibiotics for patients and the public is an additional risk for the emergence and spread of ABR.^3^, ^4^, ^5^, ^6^ However, the lack of access to effective and affordable antibiotics continues to cause more harm than the risk of ABR.^7^ Optimizing antibiotic use requires finding a balance between the need for regulation and control of excessive antibiotic use versus ensuring adequate access to those who need them. To do this effectively, we need to engage with the public and patients to bring about a culture of civil society responsibility for antibiotic use.^8^

Whilst ABR remains a global challenge, its impact is more significant in low‐ and middle‐income countries (LMICs).^9^ In the context of surgery, addressing behaviours and practices related to infection management and antibiotic use across the surgical pathway (before, during and after surgery) is key to tackling important drivers of ABR and to decreasing the burden of surgical infections globally.^10^, ^11^, ^12^ Currently, antibiotic stewardship (AS) interventions focus predominantly on prescribers, rarely considering the patient's role in antibiotic decision‐making and consumption.^13^ This is at a time when patient‐centred care, including infection‐related care, is becoming increasingly relevant.^14^ Very few studies exist on patient involvement in AS, particularly in LMIC settings.^15^ There is also a knowledge gap in healthcare professional views on patient involvement in infection‐related care. This is an important gap to explore as patient involvement is in part dependent on the willingness of healthcare staff to participate in the process.^16^, ^17^ Managing inpatient care, in ways that recognize the patient's role as a participant, remains challenging particularly in surgical pathways.^15^, ^18^ The surgical patient's involvement in pre‐ and postoperative care is crucial to optimized recovery, and yet remains difficult to achieve.

Arnstein^19^ provides a foundation for research into patient involvement, describing eight rungs of citizen participation from least to most inclusive: Manipulation, Therapy, Informing, Consultation, Placation, Partnership, Delegated power, Citizen control. Different models for types of patient involvement have been described, one of which is ‘co‐production’, or patient participation, defined as ‘user co‐delivery of professionally designed services’.^20^ At the point of care delivery, this type of involvement describes an approach where health professionals work together with patients (and/or carers) as partners to achieve optimal care. A partnership approach may not, however, be appropriate for all patients under all circumstances, and it has been argued that ‘participation should be defined by whatever level the patient is most comfortable with’.^21^

To become effective participants in their own infection‐related care, however, patients need to understand the basics and complexities of infection transmission and resistance and the role of AS to address ABR. Efforts to raise patient awareness have included written information on websites and leaflets and posters targeting healthcare users and the public, to mention a few. However, patients and the public in general may be unable to grasp the immediate threat of ABR, partly due to a lack of effective communication strategies and confusion related to the advice and information provided on the subject.^22^, ^23^, ^24^ Additionally, the social determinants of health influence clinical interactions and outcomes,^25^, ^26^ posing limitations to patients' ability to effectively participate in their own health care.^27^ In relation to infection‐related care, these factors and how they may influence health‐seeking and health‐providing behaviours remain understudied.^27^

This study aims to explore patient involvement in infection‐related care and the social and cultural factors that influence this from the perspectives of health professionals and patients and their carers in two tertiary hospitals in South Africa and India.

### 1.1 Study context

South Africa is an upper middle‐income country and India is an LMIC. Over 76% of the global antibiotic consumption between 2000 and 2010 was attributable to the BRICS countries, with India leading the group as the largest consumer of antibiotics in human health.^28^ Availability and accessibility to health systems are a challenge in LMICs. In South Africa, the majority (up to 70%) of patients access healthcare through the public sector.^29^ In India, whilst public sector healthcare services are available free of cost to patients, due to limited staff and supplies at government facilities, many seek care from the private sector (reported up to 65%), paying out‐of‐pocket.^30^ Annually, 3646 surgeries per 100,000 population are performed in India in comparison to the global estimate of 5000 surgeries per 100,000 patients.^31^ South Africa's surgical capacity has been noted to be below international requirements, with a greater concentration of available resources in urban areas.^32^, ^33^ Kerala, the state where the study site in India is located, is an atypical state with high literacy rates and better healthcare access and infrastructure compared to the rest of the country.^34^ The participating study sites were selected because despite operating in health systems with limited resources, they perform significant number of surgical interventions and have established strategies to rationalize the use of antibiotics.

Adult gastrointestinal (GI) and cardiovascular and thoracic surgery (CVTS) specialties at academic tertiary referral hospitals in South Africa (site A) and India (site B) were included in this study. Surgical specialties were selected to represent high infection and/or mortality risks.^10^, ^35^ Site A is a 950‐bed government‐funded public hospital in Cape Town, while site B is a not‐for‐profit 1350‐bed private tertiary centre in Kerala. The GI team at site B features an experienced team of GI, hepatobiliary and pancreatic surgeons. The liver transplantation team within the department is credited with 850 liver transplants and is the largest Liver Transplant Programme in Kerala. The CVTS specialty at site B is one of the busiest in the country, with over 2000 cardiac surgeries being performed annually.^36^ Both sites provide subsidized care for some patients based on income status, run established AS programmes and play key roles in AS initiatives in their respective contexts.

### 1.2 Conceptual framework

We investigated the explicit and implicit influence and participation of patients and carers in infection‐related decision‐making across the participating multidisciplinary teams. Whilst we used the definition of culture coined by Spradley: ‘the acquired knowledge people use to interpret, experience, and generate behaviours’,^37^ its application to the clinical context is built upon our existing research spanning different countries in the last 10 years.^11^, ^12^, ^38^, ^39^, ^40^, ^41^, ^42^ The existing research describes the role of hierarchies and the need for clinical autonomy in infection‐related decision‐making in inpatient settings, wherein senior doctor autonomy overrules policies.^11^, ^38^, ^40^ Recognizing the gap in knowledge, we have expanded on this research to consider the role of patients and carers in infection‐related decision‐making.^15^, ^42^

## 2 METHODS

### 2.1 Study design

Data were collected between June 2018 and November 2019 by trained researchers through nonparticipant ward round observations and semi‐structured face‐to‐face interviews with patients, patient carers and healthcare workers (HCWs). Documentary analysis of inpatient records, and the policy and guidelines on antibiotic prescribing provided contextual knowledge.

### 2.2 Nonparticipant observations

Data were gathered from general ward and intensive care unit (ICU) rounds. Four trained researchers and their trainer took notes of their observations, specifically on the following: place, the people involved, actions of participants, related activities carried out, tasks and results that participants tried to accomplish, emotions felt or expressed, the major events that occurred, the discussions that took place, who led the discussions, who acted upon identified plans. In Site B, additional data were gathered from the outpatient clinics, operation theatres and during departmental meetings. A previously used and tested data collection guide^39^ facilitated data consistency.

### 2.3 Face‐to‐face interviews

Study participants were recruited using convenience sampling and participation was voluntary, at a place and time convenient for the participant. A semistructured interview guide was used for the interviews, differentiated for patients and HCWs. In addition to this, questions that came up during observations were put forth for discussion. Interviews were conducted by the four trained researchers (two trained researchers at each site), with or without the study lead who had provided training.

### 2.4 Study participants

All HCWs involved in patient care in the surgical specialties of interest were eligible to participate. This included HCWs with different roles, experiences and expertise in the surgical teams and those from nonsurgical teams who had input into the care of surgical patients (e.g., the AS team). Patients admitted to any of the surgical specialties of interest were eligible for inclusion in the study. For the interviews, patients who were prescribed therapeutic antibiotic(s) while under the care of the surgical team(s) were invited to participate. Participants were selected using the purposive sampling technique.

### 2.5 Data analysis

Before analysis, a coding framework created by the four trained researchers and the study lead was validated through group discussions. Data from each setting were thematically analysed by researchers. Field notes and interview transcripts were analysed using the grounded theory approach—a method extensively used by the research team and published^11^—aided by NVivo 12® Pro software. Analyses of data were iterative and recursive, using constant comparison. Following analysis, the researchers discussed emerging themes for revision as required. Redundant themes were removed and other themes were collapsed or expanded as necessary. The analysis process for each study site was undertaken separately to avoid analytical bias between sites.

The different data collection methods of ward round observations, face‐to‐face interviews and HCW and patient/care interactions provided cross‐validation and triangulation of findings. To mitigate professional biases, our research team included two pharmacists, one anthropologist, one nurse, three infectious diseases specialists and five physicians from a range of surgical specialties (GI, general, cardiothoracic and emergency). The diversity of backgrounds enabled us to consider our role‐related biases and examine different perspectives throughout the analysis.

## 3 RESULTS

In site A, data were collected from 72 h of observations, including 960 episodes (659 in GI and 301 in CVTS) of bedside discussions with patients and/or their carers. Interviews were conducted with 61 HCWs and 7 patients. In site B, data were collected from 138 h of observations, including 883 episodes (399 in GI and 484 in CVTS) of bedside discussions with patients and/or their carers. Interviews were conducted with 44 HCWs and 6 patients with/without their carers.^12^ Whilst attempts were made to recruit more patients during the data collection phase of the study, we found recruitment to be challenging, as many patients, unfamiliar with participation in research, were not comfortable with providing consent to be interviewed. The observations of communication between the HCWs, patients and their carers during ward rounds provided rich insight into the boundaries of patient and carer roles and participation in infection‐related care. Analysis of data from across the study sites identified the following themes: lack of understanding of the threat of infection and ABR and the patients' positionality—both culturally and economically—and how this may influence the extent of their involvement in decision‐making.

### 3.1 A lack of understanding of the threat of infection and ABR

#### 3.1.1 Awareness of infections

While some of the patients knew that antibiotics are for treating infections, general awareness seemed to be lacking regarding the specific infection being treated (X1; Table 1). Some patients associated clinical improvement with antibiotic prescription and use, while others discussed the need for antibiotics to treat a virus (X2, X3, X4; Table 1). Prescribers also feel the demand for antibiotics from some patients in site B (X4; Table 1). Patients report a feeling of stigmatization with having an infection, with some demonstrating a lack of understanding of the processes involved in their care (X5; Table 1).

**Table 1:** Excerpts from study data (interviews and field notes).

Theme	Excerpt ID	Excerpt from data
A lack of understanding of the threat of infection and ABR	X1	‘There was no reason for the presence of germs in my blood. I was told that it was due to blood transfusion. Three persons came to give blood from my son's workplace. Then younger son's friends also came. They are not bad boys. We cannot say the germs are from their blood; or cannot say the germs are in my body … I cannot blame anybody. Any way I was given antibiotic and I had it’. Patient, CVTS, India
	X2	‘Most of the people actually they want some medicine like antibiotic … when they come for review, they will ask why I was not given an antibiotic, my wound is open and all, so the problem is there, they expect some antibiotics from us. They think that antibiotic is secure for them’. GI Surgeon, India
	X3	‘I am getting antibiotics. They are going to give me a certain type of antibiotic … the bottle is now not here … but it is a special antibiotic for that virus’. Patient, GI, South Africa
	X4	‘…there is always a feeling that, you know, if the patient becomes unwell and comes back to the ICU, many of them would ask you that is it because you have not given them an antibiotic’. GI Surgeon, India
	X5	‘In the beginning, I felt so bad; I thought it was my fault (referring to contact precaution notices). Now I see them on other doors and so, I don't feel bad anymore’. Patient, CVTS, South Africa
	X6	Senior Registrar (SR) 1 informed the patient that she can be discharged tomorrow but patient was very reluctant. SR1 suggested that he will discontinue all IV medications tomorrow and the day after the patient can be discharged to the guest house. He suggested the patient can stay there for two days and see if there is any recurrence of pain. If there is no issue, then they can go home. Patient appeared to be happy with that option. SR1 pointed out that if she stays in hospital for longer there is a chance of getting infection. Field notes, GI surgery, India
	X7	‘… that was after the operation. they gave me a tablet, yes, one or two … yes, for the pee, maybe, yes, maybe that was the antibiotic … I couldn't keep my pee in. They gave tablets because every time I have a pee0. What is that? What do you call that? Isn't that infection?’ Patient, GI Surgery, South Africa
	X8	‘If somebody is educated and aware, these are the people who would not want to use antibiotics. Even for the children, they would say that, you know, antibiotics are not good’. GI Surgeon, India
	X9	‘Well, it is not that we are going just to educate regarding the antibiotic. Obviously, it comes up in conversations, and then we take it from there. It is not like we go and counsel every patient about the antibiotic use, and in our state, I think it is not that bad, you know, compared to other states of you know India because literacy level is high, and you know even people understand that you do not need the antibiotic for everything’. GI Surgeon, India
	X10	The update mentioned that the patient had a temperature of 38.8°C the day before. Consultant 1 asks the patient if he has burning upon urinating to which the patient responded no. Consultant 2 looks at the wound and asks if the patient is on antibiotics. Consultant 1 confirms that the patient is on antibiotics and provided the name and route of administration. Consultant 2 tells the patient that he (the patient) has temperature spikes, probably due to an infection. Consultant 2 tells the patient that he is already on antibiotics but that they need to sort out the cause of the spike in temperature. Field Notes, GI Surgery, South Africa
	X11	‘After the suture removal, the surgical resident informed the carers that wound is still infected and antibiotics are being administered. He also informed that there is a suspicion of urinary tract infections and advised to call and enquire about urine culture report to change the antibiotic if needed’. Field notes, GI surgery, India
	X12	‘We do visit them and talk to them once they are shifted to the ward. Sometimes they would want to know how is it after the transplant, what is the dietary changes needs to be made. There is a dietician to direct them, but still we go and talk to them. We educate them about the infection control like they have to hand wash regularly, the bystander should wear the mask’. MSW, GI Surgery, India
Patients' positionality, both cultural and economic, may influence the extent of their involvement in decision‐making	X13	The consultant turns to the patient and asks if he knows when the next stage of his procedure will take place. The patient says that he does not know, adding lightly that he did not know there is another stage. The consultant tells the patient that he must ask questions about anything that is not clear to him (the patient)—to any one on his management team—and proceeds to explain the stages of the patient's management, what has been done so far and what may still need to be done. Field Notes, ICU, South Africa
	X14	‘The patient doesn't compel us to start the medication because they are not aware of those things. They do say that they are feeling feverish. I saw the wound sores while bathing. They don't know that the medicine needs to be started for that. They tell the doctor also that the wound was wet, there was discharge and things like that, but they don't suggest an antibiotic to give them…’. GI Staff nurse, India
	X15	‘Often, we will talk to a patient and if the patient says, “I don't want to know, just take the responsibility,” and it happens more often than not … when you get to that, it just becomes habit forming … it's the only way I have known … the profile changes completely when you are dealing with private patients because they are generally much better educated and they have much higher demands’. CVTS Surgeon, South Africa
	X16	‘Not as a rule, you would see patients saying these doctors didn't wash their hands when they examined me … I think we haven't really got to the point where the patients are litigation conscious, we don't have that culture yet. It is getting there, and it depends on the patient, some patients are more informed than others. It will change I am sure particularly in the private sector. But it is a good change, probably what we are trying to get out there. Everybody takes responsibility for infection control’. AS Staff, South Africa
	X17	‘We can't get through to the staff it feels like you are talking to a brick wall … so, maybe we should just start empowering our patients. We have designed a few patient pamphlets ourselves, and in the little pamphlet we ask, “did you ask the health care worker, did the health care worker wash his or her hands before they touched you? Were you able to remind them?” … but our patients come from poor socioeconomic backgrounds, most of our patients so they are just grateful for the care they are going to receive and we know, it is human nature, if I am going to say to you, please wash your hands before you, I am going to get your back up and so you know what I am just going to do the bare necessities here and I am going to walk away and I am not going to communicate with a patient so that is the nature, patients don't want to be victimized’. IPC Staff, South Africa
	X18	‘My husband and sons took all the decision about my care while I was in hospital. It is all their choice. They won't ask anything’. Patient, CVTS, India
	X19	Carer of a patient came in [patient not present]. The surgeon greeted him. Carer explained the patient's history to the surgeon and showed the reports of the tests done in an outside hospital. After reviewing the reports, the surgeon explained to the carer that the patient cannot be operated on due to advanced age and clinical condition. He suggested that it would be better to continue palliative care for the patient. He apologized to the carer for not being able to help them. Field notes, GI surgery outpatient clinic, India
	X20	‘I had a doubt since I am a nurse, I know normally after any surgery, they start with antibiotics, right? But I didn't see them giving any injection at all. When I asked the nurse, the nurse told that now without antibiotic it will heal, there is no need for antibiotics. On the fourth day, my daughter who is also a doctor, will be calling asking for the culture result. I too would ask but they would tell it has not come yet. I know, in our hospital if there is no outcome in 24 hours, they will give preliminary as no growth, but here even after four days, the results are not out yet. Even after the culture result came that night, they did not inform any doctor and they did not start any medications too. They did not change the antibiotic based on the growth’. Patient carer, CVTS, India
	X21	The consultant then mentions that the patient's family is coming from a coastal town approximately four hours away and that they need to talk to the family. Field Notes, CVTS, South Africa
	X22	‘We refer them all to the doctors. They are here all the time … we just say, “Doctor the patient or the family is asking questions, can you explain to them?” Then he explains to them’. Nurse, CVTS ICU, South Africa
	X23	‘If I am able to convince the patient, the patient will go back happily. If I am not able to convince the patient, well the patient will go from me to another surgeon, and then to another surgeon, ultimately to a surgeon who will actually prescribe an antibiotic and then he would be happy there, so he will go off doctor shopping’. GI Surgical Resident, India
	X24	‘I was transferred here when my funds ran out. If I had known of the wonderful care I would have received here, I would have said from the beginning, “Take me to this hospital” … There in private, it was all about the money, but here it is all about the care’. Patient, GI Surgery, South Africa
	X25	‘….so even then in some patients, who have complete financial restraints, we will have to maybe reduce the doses or take into consideration other drugs…. the other thing that happens is they will want to go to another hospital also, so probably somewhere in medical college where the medicine is free, we would recommend that’. Pharmacist, GI surgery, India
	X26	‘I think it is more a bit of, a case of, they're in a hurry, you know; like the lady that was helping me to eat—you know, and I've noticed—this morning when there was time, she had on an apron but when she came through now she didn't wear an apron, but, it is all right’. Patient, GI surgery, South Africa
	X27	‘Patients’ role is also there because some patients themselves ask the doctor, suppose they come to outpatients and they would have read up on something and they will be asking whether they should be on antibiotics. In such cases, the patient has to be reassured that they do not require an antibiotic’. GI Surgical Resident, India
	X28	‘Sometimes it is just the patient's pressure, that the patient might not feel that I am a good enough doctor in case I have not satisfied the patient's prescription as well and satisfying the patient is also a very important part of our practice. Whether we satisfy them by prescribing what they want or by convincing them that they actually do not need it. Either way the patient has to be satisfied, right, and ultimately I think the patient is satisfied. The patient wants results. So with an antibiotic or without an antibiotic, if the patient actually can get well, if that convinces the patient beyond any doubt that yes, he did not need an antibiotic, or did he need an antibiotic at that point’. GI Surgical resident, India
	X29	‘Some of the patients do tend to ask for antibiotics because they have been used to these five days of antibiotic [to be] necessary. We do tell them that we follow whatever is the standardized protocol all over the world, i.e., give prophylactic antibiotic and repeat every four hours during the surgery. Postop, unless there are signs of infection or anything, we do not give any antibiotic, in that case usually they understand. I hope they do not go back and buy it on their own’. GI Surgeon, India
	X30	‘I find it much simpler to prescribe three days of ofloxacin to somebody rather than face a litigation based on completely unscientific allegation by somebody from outside’. GI Surgeon, India
	X31	‘Patient expectations, I think defensive medicine, a genuine fear of harming the patient by withholding therapy, and using inadequate diagnostic tests. It's really hard, I'm not saying it's easy … most general practitioners in South Africa are in private practice and it's a business; so, if you don't give your patient what they want, they'll go somewhere else and your livelihood is at risk’. AS Physician, South Africa
	X32	‘It is a real problem, and in India, there is an even bigger problem out in the community, so as you know, patients who go to see a clinician, a general practitioner, usually get antibiotics. So even if it is a viral fever, they might end up getting an antibiotic … you will be surprised that some of the antibiotics that you would think three times before using even in tertiary care centers, is used very frequently [in a small hospital]’. GI Surgeon, India
	X33	‘The cost of antibiotic especially when they undergo treatment in a hospital like this [is an issue]. Many patients would not be able to afford high end antibiotics. Sometimes, we will [switch therapy] to lesser antibiotics because we have no other option’. GI Surgeon, India
	X34	‘If we had started IV antibiotic from the beginning, probably she would have improved quicker. We had initially begun treatment with intravenous antibiotic but since she could not afford it, it was changed to oral. Even though the microbiologist had suggested [stronger] antibiotics, we could not prescribe those as she wasn't willing to buy them. We prescribed a [stronger] antibiotic towards the end when we could arrange it for free from the hospital pharmacy. However, this could only be given for two days’. GI Surgeon, India
	X35	‘In India, there is no restriction on over‐the‐counter medicines, you will find the pharmacist dispensing [without prescription]. People are consuming antibiotics much more than what it used to be in the past’. GI Surgeon, India
	X36	‘Absolutely, [previous exposures to multiple antibiotic] limits our choice of antibiotics … misuse of antibiotics or over usage of antibiotics and not giving proper courses of antibiotics, well, that has caused a resistance and now we are now called the country of superbugs. It is just because anybody here, right from a registered medical practitioner to a doctor, can prescribe antibiotic; even the patient can actually go and even the pharmacists can prescribe an antibiotic and that is what has led to us you know to a problem that we have you know multidrug resistance bacteria which are not sensitive to any antibiotic’. GI Surgical Resident, India

Abbreviations: CVTS, cardiovascular and thoracic surgery; GI, gastrointestinal; ICU, intensive care unit; IPC, infection prevention and control; MSW, medical social worker.

We observed a general lack of awareness of healthcare‐associated infection (HCAI) risk among patients at site B. Patients and their carers preferred to remain in hospital as they felt they were safer there, where healthcare attention was closer than at home, despite reassurances from the surgical team that they were fit for discharge (X6; Table 1). The terms used by patients to describe infection in site A highlight how the perspectives of infection and illness differ between patients (X3, X7; Table 1). Patients referred to antibiotics as treatment for ‘viruses’ or ‘germs’ (X1, X3; Table 1).

HCWs considered the patient's socioeconomic status and/or level of education to be a factor in their understanding of antibiotic use. Surgeons at site B considered patients with higher educational qualifications to have a better understanding of antibiotic use and misuse (X8, X9; Table 1).

#### 3.1.2 HCW communication with the patient

The content and level of communication and engagement varied across the specialties. At site A, communication patterns of surgeons with patients were individualistic and depended on the communication style of individual HCWs, whereas at site B, communication patterns were department‐ or specialty‐specific. In site A, patient engagement depended on the approach of the individual senior surgeon leading the ward round and varied from a simple greeting to details inviting the patient to respond. In site B, there was consistency within specialties in the approach to patient engagement. In site B, the senior surgeons in the GI specialty took time during the rounds to speak with patients and/or their carers regarding the patient's progress and the next steps for treatment and hence the ward rounds took more time. The ward rounds led by CVTS senior surgeons were found to be much quicker, with less time spent interacting with patients and/or their carers. Across both departments, there were separate ward rounds—different from the early morning rounds—led by the surgical residents, wherein more detailed discussions were conducted with patients and/or their carers regarding any issues related to their care (care already received, care being received and care yet to be received). Across both sites, the most senior member present on the ward round was the one who engaged with the patient. This communication included discussions on their health status and plan of care (X10; Table 1). Whilst the name of the antibiotic was not mentioned in discussions with patients and/or carers, the indication for the prescribed antibiotics was generally discussed (X11; Table 1).

At site B, medical social workers (MSWs) who act as patient liaison provided psychosocial support to the patients and their families. During routine interactions with patients and carers, they also educated them on infection prevention and control (IPC) practices (X12; Table 1); however, education on ABR was not included.

### 3.2 Patients'positionality, both cultural and economic, may influence the extent of their involvement in decision‐making

#### 3.2.1 Limited patient roles

Some HCWs encouraged patients to get involved by actively engaging them in care discussions (X13; Table 1). The decision to treat and the choice of therapy for a given infection are considered to reside with the treating doctor, with patients generally not aware of opportunities that may exist for their involvement in decision‐making (X13, X14, X15; Table 1). Even if the context allowed patients to contribute or be involved in care, they may not feel empowered to do so (X16, X17; Table 1).

A greater reliance on the voice and role of the carer (often the nominated head of family or adult child of the patient) in decision‐making was observed in site B (X18, X19; Table 1), where hospital policy provides for a carer to stay with the patient. Patients' close family members played key roles in decision‐making around patient care, from the need to seek medical help to which doctor to visit and the decision to have or not have a procedure, so much so that at times, patients were excluded from this decision‐making process (X18, X19, X20; Table 1). Family members also felt to have the authority to question decisions taken by the healthcare team, stemming from the need to protect their family members (X18, X20; Table 1).

Carer involvement was more limited at site A. Healthcare teams presented updates on patient progress to each other, to the patient and to family members who may be visiting (X21; Table 1). However, no form of engagement by the patient carer or family in antibiotic discussions or in antibiotic decision‐making was observed. Nurses sometimes provided updates (infection care updates that may include information on antibiotic use) at the family's request; however, they voiced their preference to have the treating doctors do so (X22; Table 1).

#### 3.2.2 Patient's choice of healthcare provider

In the Indian healthcare systems where patients can select healthcare providers, they may have greater access to antibiotics by choosing doctors more likely to prescribe them (X23; Table 1). The patient's choice of healthcare provider is also influenced by their financial status, where patients who exhaust their ability to self‐fund care are transferred from private to public hospitals (X24; X25; Table 1).

Patients at site A expressed gratitude for the quality of care received, given their initial perceptions of care in a public healthcare environment (X24; Table 1). This awareness of the prevailing work pressures experienced by their healthcare teams as well as their gratitude for care sometimes impeded their ability to voice observed shortcomings in the care that they were receiving (X26; Table 1).

#### 3.2.3 Patient and/or carer demand for and access to antibiotics

Whilst prescribers may want to practice restraint in antibiotic prescribing, this is countered by a demand for antibiotics from patients and their families. This may be due to patients' own research on illness before seeking medical care (X27; Table 1). Prescribers in site B reported to have made efforts to educate patients and carers who demanded antibiotics (X28, X29; Table 1).

Fear of patient complaints and litigation can drive the decision to prescribe antibiotics, even though the prescriber may understand this to be irrational (X30; Table 1). The general perception, however, was that irrational prescribing is more prevalent in primary and secondary health centres than in tertiary care. Factors considered to influence inappropriate antibiotic prescribing included lack of good diagnostics and competition in community and private sectors for patients (X31; X32; Table 1).

Out‐of‐pocket expenses incurred to patients in healthcare systems that do not have universal health coverage act as an additional factor in suboptimal antibiotic prescribing. In site B, the high cost of antibiotics adds to the financial burden already placed on patients by surgery. As such, some patients may not be prescribed the most appropriate antibiotic. In some cases, where the most appropriate antibiotic is initiated, the course may not be finished due to cost constraints (X33; X34; Table 1), with implications for ABR. This was generally not the case at site A, where cost was not a factor, most likely because it was a public hospital where the financial cost of care incurred to patients was minimal.

The unregulated access to and consumption of antibiotics before presentation at the hospital were recognized by participants in site B as factors in the development of ABR (X35; X36; Table 1).

## 4 DISCUSSION

This study explored the inpatient's influence and participation in infection‐related care in surgical specialties in India and South Africa. The cultural differences are manifested in how care is provided and the extent to which patients and carers are involved in decision‐making. The patients' positionality, both economic and cultural, may influence their engagement with and expectations of care, including infection‐related care and antibiotic use. Missed opportunities for effective patient engagement in AS and IPC remain in patient pathways. This is an important gap, given that infection is one of the unintended consequences of surgery,^43^ especially in LMICs, where the burden of surgical infections is higher.^44^ In health care, effective communication is key to positive patient outcomes.^41^ This communication occurs at different levels with HCWs, patients, carers and the public. The communication needs of these different groups may vary. Patients bring their own beliefs, which need to be understood before they can be changed or influenced. While patient engagement could be affected by various clinical‐ and administration‐related pressures, some senior surgeons, regardless of these, consistently engaged the patient in discussions related to their care, including infection care decisions.^41^

Our data show that patients do not necessarily associate hospitals with infection. We need to go beyond the assumption that patient, carer and public education alone will address the identified gaps in IPC and AS and that it will foster optimized practices.^42^ The identified gaps can be addressed, perhaps by using those around the patient with additional specific roles in relation to patient education and engagement. In site B, MSWs, identified to be closely engaged in patient interactions, can provide pre‐ and postsurgical infection care advice and training to facilitate better patient participation in infection care.

Opportunities exist in targeting the involvement of HCWs in patient engagement, and participation in infection care and AS. HCWs themselves may also benefit from context‐appropriate communication skills to effectively engage patients. Depending on the context, patient carers also need to be engaged in interventions. Opportunities for patient and HCW engagement can provide learning, for both healthcare providers and recipients, on the effective means and outcomes of such engagement.

Table 2 presents recommendations for optimizing inpatients' and their carers' involvement in antibiotic decision‐making. Implementation of patient‐centred interventions has demonstrated improvements in short‐term knowledge of infections and antibiotic therapy among participants.^45^ Patients have been known to influence antibiotic prescribing,^42^, ^46^ and co‐design of AS‐ and ABR‐ focused interventions with patients may help influence attitudes and behaviours in relation to antibiotic consumption. Such efforts will also support evolving patient roles from passive recipients to active participants in care.

**Table 2:** Recommendations for optimizing patient involvement in antibiotic decision‐making.

Recommendation	Description
Efforts to check patient needs and understanding of information provided	Patients bring lay knowledge and expertise to their own care, which needs to be understood and acknowledged. Patient education needs to go with checks for patient comprehension, to assist with healthcare worker understanding where further information needs to be provided.
Identifying champions for patient‐centred antimicrobial stewardship communication	Doctors may spend limited time with patients on the ward and nurses may be limited by a low nurse to patient ratio. It would be beneficial to identify a champion for communicating with patients regarding their care. Medical Social Workers in India, who have a key role to play in patient communication, are an example. Opportunity may exist to utilize community health workers (CHWs) or home‐based carers for such roles in South Africa. CHWs are already involved in medical specialties, especially in primary healthcare centres, where they assist with care communication and support of the patient in HIV and/or tuberculosis care.^55^
Improving education to patient and carers on healthcare‐associated infections, antibiotic use and antibiotic resistance	Evidence‐based educational materials should be prepared for patient and carer education. This education could be delivered through one‐to‐one counselling, leaflets or pamphlets or other suitable means. The materials should be concise, contextually appropriate and in a language devoid of medical jargon that the patient/carer can easily understand.
Identifying role for pharmacists and nurses in providing one‐to‐one education for patients	A one‐to‐one education/counselling session may be beneficial for patient education. This session should take place ideally at a time that is convenient for the patient and should also be flexible as it may need to be repeated over time as needed. There may be opportunity for expanding the role of pharmacists and nurses in patient education. Limitations in resources (funding and time constraints) pose challenges to this and will need to be addressed.

Individualistic or not, the decision‐making in one's health is heavily influenced by the society and its perspectives on health.^47^ In different cultures, positionality of individuals within the family and wider society can determine health‐seeking behaviours and the individual's level of involvement in relation to his or her own care needs.^48^, ^49^ The role of close family members and/or carers in patient care differed across the two sites. In site B, carer engagement is supported, first, by hospital policy that mandates their presence and involvement in patient care. Second, there was a paternalistic practice of protecting the patient from what was considered sensitive information in relation to their own health, thereby excluding them from decisions about their own care, especially for patients with serious illnesses, while the carer took charge. Carers also play key roles in inpatient and post‐discharge infection‐related care.^42^ This may be a wider reflection of the cultural norms in India, where the wider family support network is more depended upon in health care.

A key point to consider is that patients will have their own knowledge and lay beliefs, which may influence their behaviours and expectations of care received,^42^, ^50^ including expectations for being prescribed antibiotics. The prescribers' accounts and experiences confirm this, particularly their fear of patients choosing a different prescriber who might be more willing to comply to the patient's demand for antibiotic prescriptions. This creates competition among doctors and hospitals, especially in the case of private hospitals, and can act as a perverse incentive to prescribe antibiotics, as described in a previous study among formal and informal health providers in West Bengal, India.^51^ In pluralistic health systems, where patients choose from a range of formal and informal healthcare providers, and the opportunity for self‐medication is higher,^52^ policy and regulation alone will not address the overuse of antibiotics. Such self‐medication practices may have implications for ABR, for instance, where self‐funding patients may be unable to afford full antibiotic courses. There needs to be sustainable engagement within civil society and the public as well as amongst HCWs on the threat of ABR, underpinned by an understanding of the context and specific patient/public behaviours that drive ABR, to bring about effective change in collective behaviours.^8^

In health care, current opportunities for meaningful and effective communication between patients and healthcare providers remain limited, particularly around IPC and AS.^15^ Active engagement with and participation of patients in their care decision‐making is critical, particularly in countries where lack of adequate universal healthcare means that patients use out‐of‐pocket expenses to fund healthcare needs. Inappropriate antibiotic use may also stem from their over‐the‐counter access, especially where antibiotic procurement cost is significantly less than that associated with a doctor's consultation or hospital admission. Literature on antibiotic self‐medication practices in an urban population in Kerala showed that 3.31% of 775 adult participants reported antibiotic self‐medication, of which 36% were procured using doctors' previous prescriptions, with convenience as the major reason for self‐medication.^53^ Other states in India with lower literacy rates report higher levels of over‐the‐counter antibiotic sales.^53^ A study from the UK investigated the educational level of older patients and their access to healthcare, highlighting that patients with more education navigate the health system and access services more effectively.^54^ HCWs in Site B also associated higher education levels with better understanding of antibiotic‐related decision‐making and use and vice versa.

Our study has limitations. It was conducted in a public hospital in South Africa and a private hospital in India. Both study sites are atypical hospitals at the forefront of implementing AS interventions and are not necessarily reflective of practices across other hospitals in each country. In addition, while the study focused on patient and carer involvement in infection care, HCW interactions were critical to providing insight and contextualization. This work presents important information on the extent of the surgical patient's involvement in antibiotic decision‐making and identifies opportunities for a more patient‐centred AS engagement. Longitudinal data collection also facilitated multiple observations, reducing the likelihood of the Hawthorne effect. In addition, the application of a data collection guide, multiple data sources and researcher reflexivity helped to minimize subjectivity in the data collection process and to validate the findings.

## 5 CONCLUSION

This study has provided new insight into surgical inpatients' involvement in infection‐related care, including AS, across two diverse settings. To have a valuable role in AS and make informed decisions related to their care, a better understanding and channelling of the knowledge and experiences that patients and carers bring to their own care needs is crucial. The universal patient‐centred approach to care, modelled through an individualistic lens, may not be responsive to the cultural determinants of health and ABR in settings like India, where a community of individuals connected to the patient has a voice in patient care with greater access to and demand for antibiotics. More effort is required to fully integrate and channel patient and carer experiences and outlooks in initiatives to address ABR, especially as it relates to the demand for and access to antibiotics.

## AUTHOR CONTRIBUTIONS

This is a multicentre study involving multiple surgical departments at each site. All the authors represent either the research team or the surgical team. All the authors have critically reviewed the manuscript and have approved the final version to be published.

## ASPIRES Co‐Investigators

Andy Leather, Raheelah Ahmad, Nick Sevdalis, Ewan Ferlie, Gabriel Birgand.

## CONFLICT OF INTEREST STATEMENT

The authors declare no conflicts of interest.

## ETHICS STATEMENT

Ethical approval was obtained from both the study sites (Ref: 499/2018 and IEC‐AIMS‐2018‐INF.CONT‐005A). Prior to study commencement, the surgical teams were sensitized to the study and their approval was sought and obtained. Buy‐in from the surgical leads eased access to the teams. For observations, researchers obtained group consent from the specialty leads and verbal consent from participants. Posters were put up in wards to make participants aware of this study taking place in the ward. Written informed consent was obtained from participants before each interview. All participant‐identifiable data were anonymized before data analysis.

## Data Availability

Data on which this publication is based are available via a secure server. Access to the data can be provided upon reasonable request. The data that support the findings of this study are available on request from the corresponding author. The data are not publicly available due to privacy or ethical restrictions.
